# Evaluating Extracellular Matrix influence on adherent cell signaling by Cold Trypsin Phosphorylation-specific Flow Cytometry

**DOI:** 10.1186/1471-2121-14-36

**Published:** 2013-08-19

**Authors:** Iren Abrahamsen, James B Lorens

**Affiliations:** 1 Department of Biomedicine, University of Bergen, Jonas Lies Vei 91, 5009 Bergen, Norway

**Keywords:** Flow cytometry, Phosphorylation, Cell signaling, Extracellular matrix

## Abstract

**Background:**

Tissue microenvironments comprise different extracellular matrix (ECM) proteins that regulate cellular responsiveness to growth factors. In vitro culture of adherent cells on ECM-coated substrata is commonly used to study microenvironmental influence on specific cell signaling responses. Phosphorylation-specific flow cytometry can be utilized to quantify intracellular phosphorylation-dependent signaling events in single cells. However this approach necessitates trypsinization of adherent cells to accommodate flow cytometric analysis. Trypsin is a potent activator of cell signaling and can obscure signal transduction events induced by other factors.

**Results:**

To address this we developed a cold trypsin-phosphorylation-specific flow cytometry protocol, where adherent cells are prepared for flow cytometric analysis on ice (~0°C), a temperature where trypsin retains activity but where intracellular kinases are inactive. We show that this straightforward approach can be used to quantify intracellular pERK levels in single adherent primary human vascular smooth muscle cells grown on different ECM.

**Conclusions:**

Exploiting the limited temperature dependence of trypsin facilitated development of a generally applicable phosphorylation-specific flow cytometry method for analysis of adherent cell types including primary patient derived cells. We demonstrate the utility of cold trypsin-phosphorylation-specific flow cytometry analysis of cell signaling to measure microenvironmental influence in single adherent cells.

## Background

The signaling pathways that drive different normal and pathological cellular responses are controlled by microenvironmental cues, including cell-cell interactions, ECM-integrin engagement and activation of receptor tyrosine kinases by growth factors. This intracellular signal transduction creates unique response-dependent epitopes that can be detected by specific antibodies providing the opportunity to measure signaling events by various immuno-techniques. Phosphorylation-specific flow cytometry enables measurement of the phosphorylation status of multiple intracellular signaling proteins at a single-cell level [1-3]. However, the laminar fluidic flow systems of standard flow cytometers require that cells are in suspension and a near-spherical configuration for effective analysis. To allow the adaptation of phosphorylation-specific flow cytometry approach to study primary adherent human cells, we took advantage of the broad temperature-independent activity of trypsin to allow proteolytic release of single adherent cells at low temperatures. We show that cold-trypsin phosphorylation-specific flow cytometry analysis can be used to quantify phosphorylated ERK (pERK) levels in single human pulmonary artery-derived smooth muscle cells (PaSMC) exposed to different ECM proteins.

## Methods

### Cold trypsin phosphorylation-specific flow cytometry analysis of adherent cells

Low passage (<10) PaSMCs (Lonza, Cologne, Germany) were grown at 37°C, 5% CO_2_ in human fibroblast growth factor (hFGF)-supplemented smooth muscle growth medium-2 (SmGM-2; Lonza, Cologne, Germany) on ECM protein coated tissue culture plates until about 80% confluence. Tissue culture plates were coated by passive adsorption with collagen I (0.1 mg/ml), fibronectin (15 μg/ml) and laminin (15 μg/ml) (Sigma-Aldrich, St. Louis, MO, USA). PaSMC were serum starved overnight prior to stimulation with 50 nM phorbol-12-myristate-13-acetate (PMA; Sigma-Aldrich, St. Louis, MO, USA) for 5 minutes or 20% fetal bovine serum (FBS; PAA Laboratories, Pasching, Austria) for 15 minutes. The cells were placed on ice immediately after stimulation to quench cell signaling. The cells were then washed twice with ice cold phosphate buffered saline (PBS; Sigma-Aldrich) before adding ice cold 0.5% Trypsin-Ethylenediaminetetraacetic acid (EDTA; Sigma-Aldrich). Culture plates were kept on ice and monitored by microscopy until the cells had completely rounded up and detached from the cell culture dish (approximately 25 minutes). Alternatively, cells were detached with 0.25% Trypsin-EDTA at 37°C for 2 minutes. A 16% paraformaldehyde (PFA) solution (Electron Microscopy Sciences, Hatfield, PA, USA) was added directly to the single cell suspension to obtain a final concentration of 1.6% PFA. The cells were incubated in the fixative for 20 minutes at room temperature. The cells were thereafter washed with ice cold PBS and pelleted before they were permeabilized in 100% ice-cold methanol (Sigma-Aldrich) overnight at 4°C [2,3]. To stain the cells for flow cytometry, the samples were first centrifuged at 500 × g for 5 minutes to remove the methanol and then washed with FACS buffer (1% BSA in PBS). Cells were then stained with rabbit anti-p44/42 MAPK (ERK1/2) antibody (#4695 Cell Signaling, Danvers, MA, USA) at a dilution of 1:1000 for 30 minutes at room temperature. The cell suspension was then washed with FACS buffer before goat anti-rabbit IgG-Alexa Fluor 647 (A-21244, Invitrogen; Life Technologies, Grand Island, NY, USA) was added at a concentration of 1:2500 (0.8 μg/mL) as a secondary antibody. The cells were incubated for 1 hour in darkness at room temperature before being washed once more with FACS buffer. Samples were run on a BD Accuri C6 (BD Biosciences, San Jose, CA, USA) equipped with 488 nm and 640 nm lasers at the University of Bergen Flow Cytometry Core Facility. Daily quality control is performed on the instrument using 8- and 6- peak bead sets. The standard bandpass filter used for Alexa Fluor 647: 675/25 Alexa fluor 647 (excitation max: 640 nm, emission max: 668 nm). A minimum of 10,000 events was collected for each sample. The gating strategy comprised a standard FSC/SSC live cell gate, followed by a FSC-A/FSC-H single cell gate to minimize inclusion of debris, dead cells and aggregates in the analysis of Alexa Fluor 647 fluorescence. Alternatively fixable live/dead dyes may be added to prior to PFA treatment to enhance discrimination of dead cells in the analysis. Flow data analysis and median fluorescence intensity (MFI) calculations were performed using FlowJo (TreeStar Inc., Ashland, OR, USA). All fluorescence values were corrected by subtracting the MFI value of the secondary only goat anti-rabbit IgG-Alexa Fluor 647 staining. Cell viability of trypsinization protocols was evaluated by propidium iodide exclusion (1 ug/ml for 5 minutes, room temperature) and flow cytometry analysis (BD FACS Aria II, 532 nm laser; 610/20 BP filter). No difference in the percentage of dead cells within the PI-positive gate (<3%) was observed following either warm or cold trypsinization of PaSMC.

### Western blot analysis

PaSMC were grown in ECM protein coated 10 cm cell culture plates until 80% confluence, serum starved and treated with 50 nM PMA for 5 minutes or 20% FBS for 15 minutes at 37°C, 5% CO_2_. After stimulation the cells were washed twice with ice cold PBS before 300 μL ice-cold Nonidet-P40 (NP40) lysis buffer was added and cells were scraped off the dish using a cell scraper and transferred to an eppendorf tube and kept on ice for 20 minutes. The samples were sonicated 3 times for 10 seconds and then centrifuged at 16,000 × g for 5 minutes at 4°C. Supernatants were transferred to a fresh tube and stored at -20°C. The protein concentration was then determined by BCA Protein Assay Kit (Thermo Fisher Scientific, Waltham, MA, USA). XT Sample buffer (Bio Rad, Hercules, CA, USA) was added to the samples and heated at 95°C for 10 minutes, cooled down and loaded onto NuPAGE 10% Bis-Tris gels (Invitrogen, Carlsbad, CA, USA), and run for 50 minutes at 200 Volts in NuPAGE MOPS SDS Running Buffer (Invitrogen). Proteins were transferred to a polyvinylidene fluoride (PVDF) transfer membrane (GE Healthcare, Little Chalfont, Buckinghamshire, UK) for 1 hour at 100 Volts. The membrane was blocked in TBS/T (0.1% Tween 20) with 5% (weight/volume) dried skimmed milk powder. After blocking, the membrane was washed 4 times for 5 minutes in TBS/T. The membrane was then incubated with anti-pERK primary antibody (p44/42 MAPK antibody 4695; Cell Signaling) at a dilution of 1:2000. The primary antibody was added in TBS/T with 5% (w/v) BSA and incubated over night at 4°C. The membrane was then washed 4 times for 5 minutes in TBS/T before staining with Goat Anti-Rabbit IgG (H+L)-HRP Conjugate 170–6515 (Bio-Rad) secondary antibody at a dilution of 1:10,000. The membrane was incubated with secondary antibody in TBS/T with 5% (w/v) dried skimmed milk powder for 1 hour at room temperature before being washed again, 4 times for 5 minutes with TBS/T. Loading control was performed using monoclonal mouse Anti-Human Smooth Muscle Actin Clone 1A4 (Dako, Glostrup, Denmark) with Goat Anti-Mouse IgG (H+L)-HRP Conjugate 170–6516 (Bio-Rad) as secondary antibody. The membrane was developed in a Chemi Doc XRS (Bio Rad) using Pierce ECL Western Blotting substrate (Thermo Fisher Scientific). Quantification of Western blot bands was conducted using the ImageLab 3.0 software (BioRad). Mean pixel intensity was measured for each band, background was subtracted and samples were normalized to beta-actin.

## Results and discussion

### Quantifying pERK in single adherent cells by phosphorylation-specific flow cytometry

Flow cytometry of adherent cells is routinely carried out by first detaching cells from tissue culture dishes or dissociating intact tissues by treating with the serine protease trypsin. Trypsin is known to activate cell signaling via the protease-activator receptor 2 (PAR2), a seven transmembrane-spanning domain G protein-coupled receptor, by cleaving the amino-terminal exodomain [4]. PAR2 activation stimulates MAP kinase pathway activity in epithelial and smooth muscle cells [5,6]. Trypsin activation of PAR2 is also shown to activate MAP phosphatase activity and attenuate pERK level induction by other receptors [6,7]. Hence, trypsin can lead to both potentiated and attenuated pERK levels induced by other receptors.

**Figure 1:**
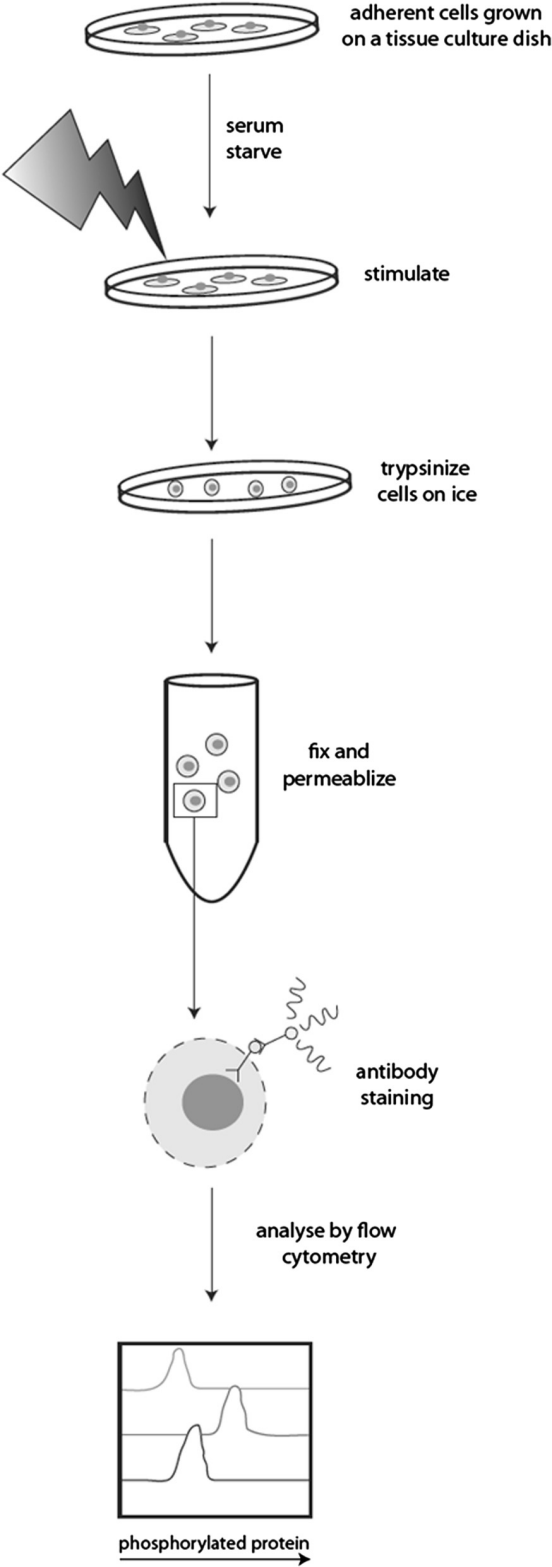
**Schematic of the cold trypsin phosphorylation-specific flow cytometry approach.** Cells grown on cell culture dishes at 37°C are treated with activators for the required time and then immediately placed on ice to quench further cell signaling. Ice cold trypsin is added to the cell culture plates and kept on ice during the trypsinization process in order to minimize intracellular signaling. The cells are then harvested, fixed and permeablized prior to staining with optimized phospho-specific primary antibodies and fluorophore-conjugated secondary reagents. Stained cells are analyzed by flow cytometry to quantify single cell fluorescence values corresponding to phosphorylated levels of intracellular signaling proteins.

**Figure 2:**
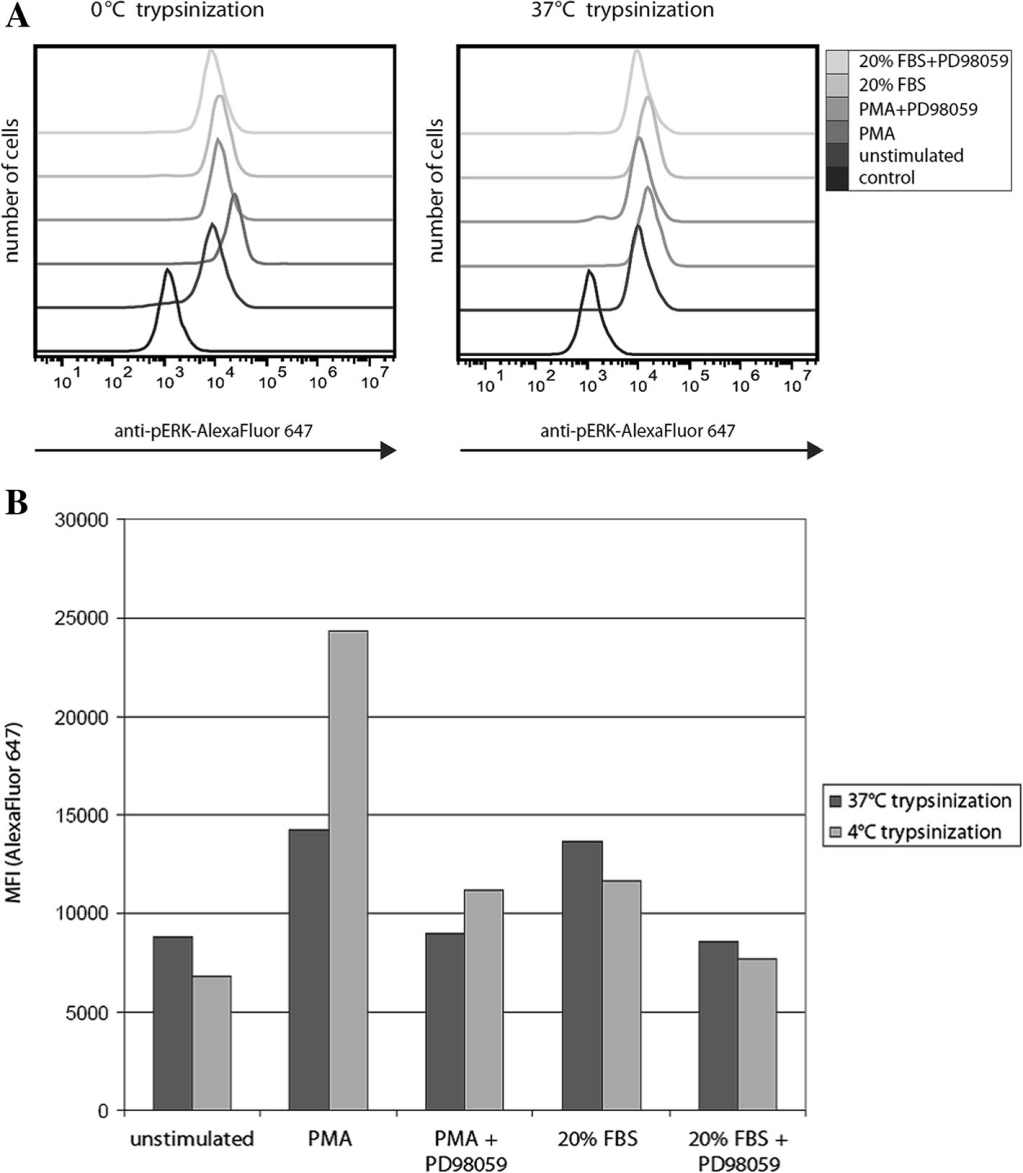
**pERK levels are affected by post-treatment trypsinization at different temperatures.** PaSMC cultures were stimulated with PMA 50 nM for 5 minutes or 20% FBS for 15 minutes at 37°C, 5% CO_2_ and prepared for phosphorylation-specific flow cytometry analysis by trypsinization at 37°C or 0°C. Cells were pretreated with the MEK1 inhibitor PD98059 at 30 μM for 3 hours to inhibit pERK activation. **A**: Flow cytometry analysis of pERK levels in PMA- and serum-treated PaSMC. Rabbit anti-pERK/goat-anti-rabbit-Alexa Fluor 647 secondary antibody was quantified (10,000 events) and plotted as overlaid histograms of fluorescence versus number of cells. **B**: Median fluorescence intensity (MFI) measured for PMA- and serum-induced pERK levels following post-treatment trypsinization at 37°C or 0°C. Trypsinization at 37°C both increased the basal pERK levels and attenuated the activation of pERK by PMA. Control: Goat-anti-rabbit Alexa Fluor 647 secondary only. (Representative of three independent experiments).

We reasoned that adherent cells could be prepared for phosphorylation-specific flow cytometry using a cold trypsin solution (Figure 1). Trypsin remains nearly 50% active at 4°C, a temperature where intracellular kinases lack activity [8]. Trypsinization of PaSMC on ice (~0°C) did not adversely affect cell viability (data not shown), consistent with a previous report [9]. Semiconfluent low passage PaSMC cultures were stimulated with PMA at 50 nM for 5 minutes, or with 20% FBS for 15 minutes and prepared for phosphorylation-specific flow cytometry analysis by trypsinization at 37°C or on ice. Cells were kept on ice during the cold trypsin protocol in order to quench cell signaling. PaSMC detached within 25 minutes on ice and within approximately 2 minutes at 37°C. Detached PaSMC were collected, fixed with paraformaldehyde, permeabilized with methanol and stained with anti-pERK1/2 (phospho-Thr202/Tyr204) antibody. Stained cell suspensions were analyzed by flow cytometry to determine pERK levels. In preliminary experiments we noted variation in pERK levels following trypsinization at 37°C in unstimulated cell cultures, while basal pERK levels remained low following trypsinization at on ice (data not shown). This is in part due to the rapid detachment of PaSMC at 37°C. As shown in Figure 2, PaSMC treated with 50 nM PMA and recovered by trypsin treatment at 37°C showed a 1.6-fold increase in pERK (MFI=14210) as compared to the unstimulated cells (MFI=8844). In contrast, pERK levels in cells recovered by cold trypsin showed a 3.6-fold increase following treatment with PMA (unstimulated, MFI=6806; PMA treated, MFI=24341). Relative pERK activation was 1.5-fold increase by 20% FBS treatment when trypsinized at 37°C (unstimulated, MFI=8844; serum treated, MFI=13678) and 1.7-fold increase by 20% FBS treatment with cold trypsin (unstimulated, MFI=6806; serum treated, MFI=11713). In order to verify the specificity of the anti-phosphorylated ERK signal detected by phosphorylation-specific flow cytometry, cells were pretreated with an inhibitor of the upstream MAP kinase MEK1 (PD98059) at 30 μM for 3 hours. Pretreatment with PD98059 inhibited pERK activation in PaSMCs stimulated with PMA or serum stimulation (Figure 2). Collectively, these results indicate that the cold trypsin phosphorylation-specific flow cytometry protocol is a preferable approach to measure cell signaling changes in adherent cells.

### Activation of the MAP kinase pathway is regulated by ECM

Vascular smooth muscle cells (vSMC) are normally surrounded by a laminin-rich basal lamina. During homeostatic or pathological vascular remodeling, vSMCs are exposed to dynamic changes in the local ECM, including loss of basement membrane and synthesis of new ECM components such as fibronectin, that can lead to vSMC proliferation, a major contributor to restenosis, vein graft thickening and atherosclerosis [10-12]. The laminin-rich basal lamina suppresses growth and promotes vSMC differentiation, while fibronectin transitional matrix deposited during vascular remodeling stimulate growth [13].

**Figure 3:**
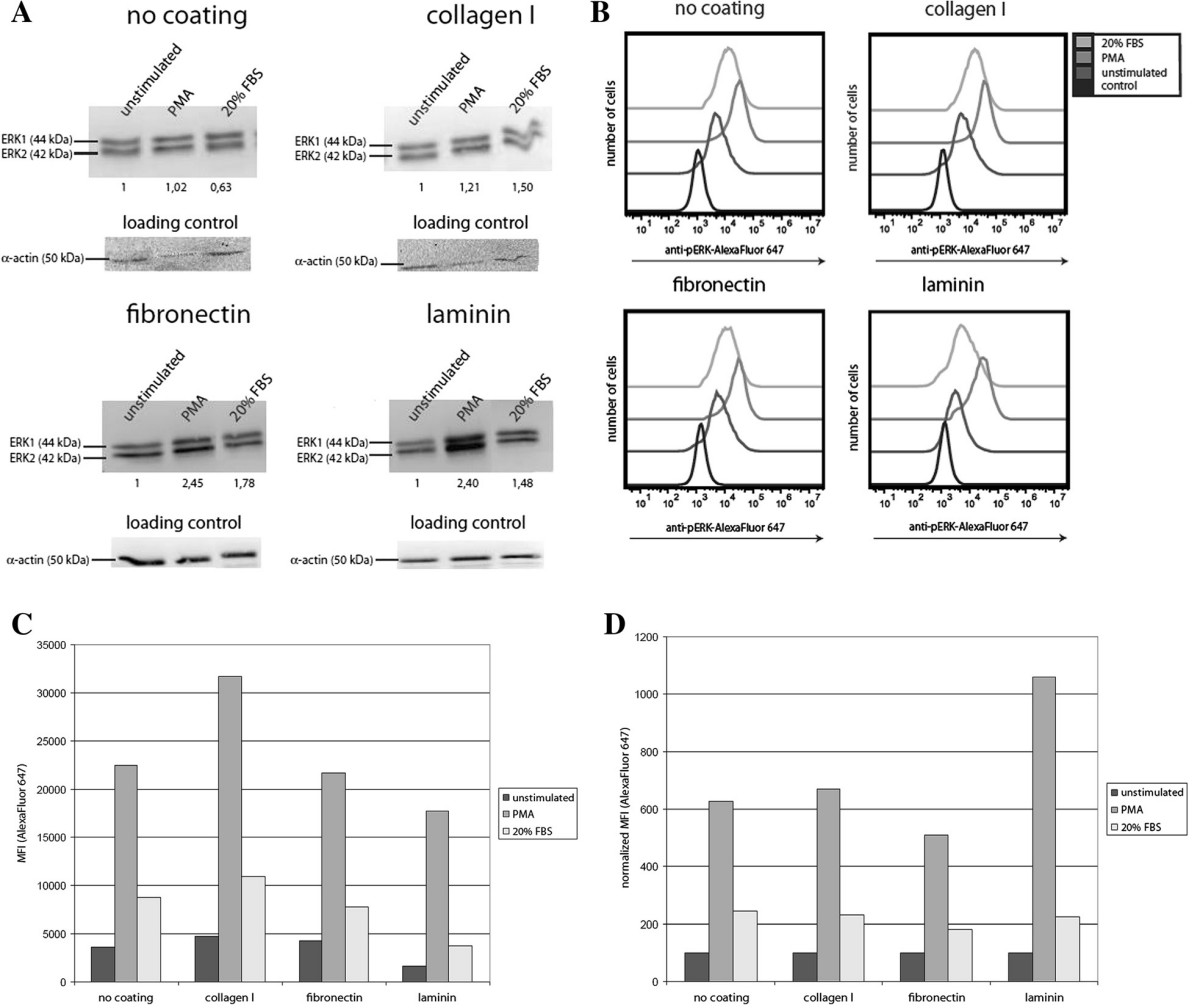
**PaSMC adherence to extracellular matrix regulates the MAP kinase pathway.** Semiconfluent PaSMC cultured on collagen I, fibronectin and laminin and analyzed by Western blot or cold trypsin phosphorylation-specific flow cytometry to measure changes in pERK following stimulation with PMA and serum. **A**: Western blot analysis of pERK1/2 levels in PaSMC lysates from PMA and serum treated cells cultured on collagen I, fibronectin and laminin. Anti-smooth muscle actin is shown as a loading control. Normalized mean pixel intensity values for the pERK1/2 bands are indicated. **B**: Analysis of anti-pERK levels by cold trypsin phosphorylation-specific flow cytometry of PMA and serum treated PaSMC cultured on uncoated and collagen I, fibronectin and laminin-coated plates revealed a uniform increases in pERK activation. Plotted as overlaid histograms of fluorescence versus number of cells. **C**: Median fluorescence intensity (MFI) of anti-pERK levels determined by cold trypsin phosphorylation-specific flow cytometry of PMA and serum treated PaSMC cultured on collagen I, fibronectin and laminin. **D**: Plot of MFI values normalized to unstimulated PaSMC cultures on uncoated and collagen I, fibronectin and laminin-coated plates. Control: Goat-anti-rabbit Alexa Fluor 647 secondary only. (Representative of three independent experiments).

To address the effect of different ECM proteins on MAP kinase signaling in vSMC, we cultured PaSMCs on tissue culture plates coated with collagen I, fibronectin or laminin with growth factor (FGF) and serum supplemented culture medium. Semiconfluent PaSMC cultures were starved overnight and stimulated with PMA or serum. Western blot analysis of PaSMC lysates (Figure 3A) showed relative increases in both pERK1/2 bands at 42 and 44 kDa, following treatment with PMA or serum. In order to quantify these ECM-induced changes in MAP kinase pathway activation, we conducted the cold trypsin phosphorylation-specific flow cytometry analysis to measure pERK levels. Histogram plots of the anti-pERK fluorescence (Figure 3B) revealed a uniform increase in pERK activation in PMA and serum treated PaSMCs cultured on different ECM. Basal pERK levels in PaSMCs cultured on laminin were 2.8 and 2.5 fold less than PaSMC cultured on collagen I or fibronectin respectively (Figure 3C). Treatment with PMA or serum strongly activated pERK under all conditions. Compared to unstimulated cells (MFI=3576), pERK levels in cells cultured on tissue culture dishes with no coating were upregulated 6.3 and 2.3 fold when treated with PMA (MFI=23700) and serum (MFI=8808) respectively. For cells growing on collagen I, treatment with PMA and serum upregulated pERK levels 6.7 and 2.3 fold (unstimulated, MFI=4733; PMA treated, MFI=31758; serum treated, MFI=10906). Cells on fibronectin were upregulated 5.1 and 1.8 fold when treated with PMA and serum respectively (unstimulated, MFI=4249; PMA treated, MFI=21739; serum treated, MFI=7771), while in cells grown on laminin-coated dishes the upregulation of pERK levels were 10.6 and 2.3 fold when treated with PMA (MFI=17690) or serum (MFI=3775) compared to the unstimulated cells (MFI=1668). PaSMC cells cultured on collagen and fibronectin display a nearly identical pERK response profile to that of cells cultured on uncoated plates when normalized against unstimulated cells, while cells cultured on laminin displayed an increased relative pERK responsiveness to PMA stimulation (Figure 3D).

## Conclusions

Exploiting the limited temperature dependence of trypsin facilitated development of a generally applicable phosphorylation-specific flow cytometry method facilitating cell signaling analysis in adherent cell types including primary patient derived cells. We demonstrate the utility of cold trypsin-phosphorylation-specific flow cytometry analysis of cell signaling to measure ECM influence in single adherent vSMC. Our results indicate that vSMC adherence to laminin reduces basal pERK levels consistent with suppressed growth, but maintain responsiveness to serum and PKC-dependent signaling.

Flow cytometry analysis offers an attractive alternative to other approaches to multiparametric measurement of phosphorylation-dependent cell signaling events in adherent cells at single cell resolution, such as fluorescence scanning of arrayed cells or high content microscopy-based systems [14]*.* In particular, phosphorylation-specific flow cytometry offers superior quantification of phospho-epitopes detected by immunofluorescence [2,3]. As this sensitivity is achieved at the expense of cell morphological information, an integration of complementary flow cytometry and microscopy approaches is optimal.

## Competing interest

The authors declared that they have no competing interests.

## Authors’ contributions

IA performed experiments, data analysis and interpretation and contributed to preparation of the manuscript; JBL conducted data analysis, interpreted data and writing of the manuscript. Both authors read and approved the final manuscript.
